# Disease severity, arrhythmogenesis, and fibrosis are related to longer action potentials in tetralogy of Fallot

**DOI:** 10.1007/s00392-023-02288-z

**Published:** 2023-09-19

**Authors:** Hannah E. Fürniss, Eike M. Wülfers, Pia Iaconianni, Ursula Ravens, Johannes Kroll, Brigitte Stiller, Peter Kohl, Eva A. Rog-Zielinska, Rémi Peyronnet

**Affiliations:** 1 Institute for Experimental Cardiovascular Medicine, Faculty of Medicine, University Heart Center Freiburg-Bad Krozingen, University of Freiburg, Freiburg, Germany; 2 Department of Congenital Heart Defects and Pediatric Cardiology, Faculty of Medicine, University Heart Center Freiburg-Bad Krozingen, University of Freiburg, Mathildenstr. 1, 79106 Freiburg, Germany; 3 Department of Physics and Astronomy, Ghent University, Ghent, Belgium; 4 Department of Cardiovascular Surgery, Faculty of Medicine, University Heart Center Freiburg-Bad Krozingen, University of Freiburg, Freiburg, Germany; 5 Signaling Research Centers BIOSS and CIBSS, University of Freiburg, Freiburg, Germany

**Keywords:** Congenital heart disease, Arrhythmias, Fibrosis, Electrophysiology

## Abstract

**Background:**

Arrhythmias may originate from surgically unaffected right ventricular (RV) regions in patients with tetralogy of Fallot (TOF). We aimed to investigate action potential (AP) remodelling and arrhythmia susceptibility in RV myocardium of patients with repaired and with unrepaired TOF, identify possible correlations with clinical phenotype and myocardial fibrosis, and compare findings with data from patients with atrial septal defect (ASD), a less severe congenital heart disease.

**Methods:**

Intracellular AP were recorded ex vivo in RV outflow tract samples from 22 TOF and three ASD patients. Arrhythmias were provoked by superfusion with solutions containing reduced potassium and barium chloride, or isoprenaline. Myocardial fibrosis was quantified histologically and associations between clinical phenotype, AP shape, tissue arrhythmia propensity, and fibrosis were examined.

**Results:**

Electrophysiological abnormalities (arrhythmias, AP duration [APD] alternans, impaired APD shortening at increased stimulation frequencies) were generally present in TOF tissue, even from infants, but rare or absent in ASD samples. More severely diseased and acyanotic patients, pronounced tissue susceptibility to arrhythmogenesis, and greater fibrosis extent were associated with longer APD. In contrast, APD was shorter in tissue from patients with pre-operative cyanosis. Increased fibrosis and repaired-TOF status were linked to tissue arrhythmia inducibility.

**Conclusions:**

Functional and structural tissue remodelling may explain arrhythmic activity in TOF patients, even at a very young age. Surprisingly, clinical acyanosis appears to be associated with more severe arrhythmogenic remodelling. Further research into the clinical drivers of structural and electrical myocardial alterations, and the relation between them, is needed to identify predictive factors for patients at risk.

**Graphical Abstract:**

Central illustration: summary diagram of essential study results. Note that not all results are depicted here. For more detail, see text. *APA* action potential amplitude, *APD* action potential duration, *AUC* area under the curve, *TOF* tetralogy of Fallot.

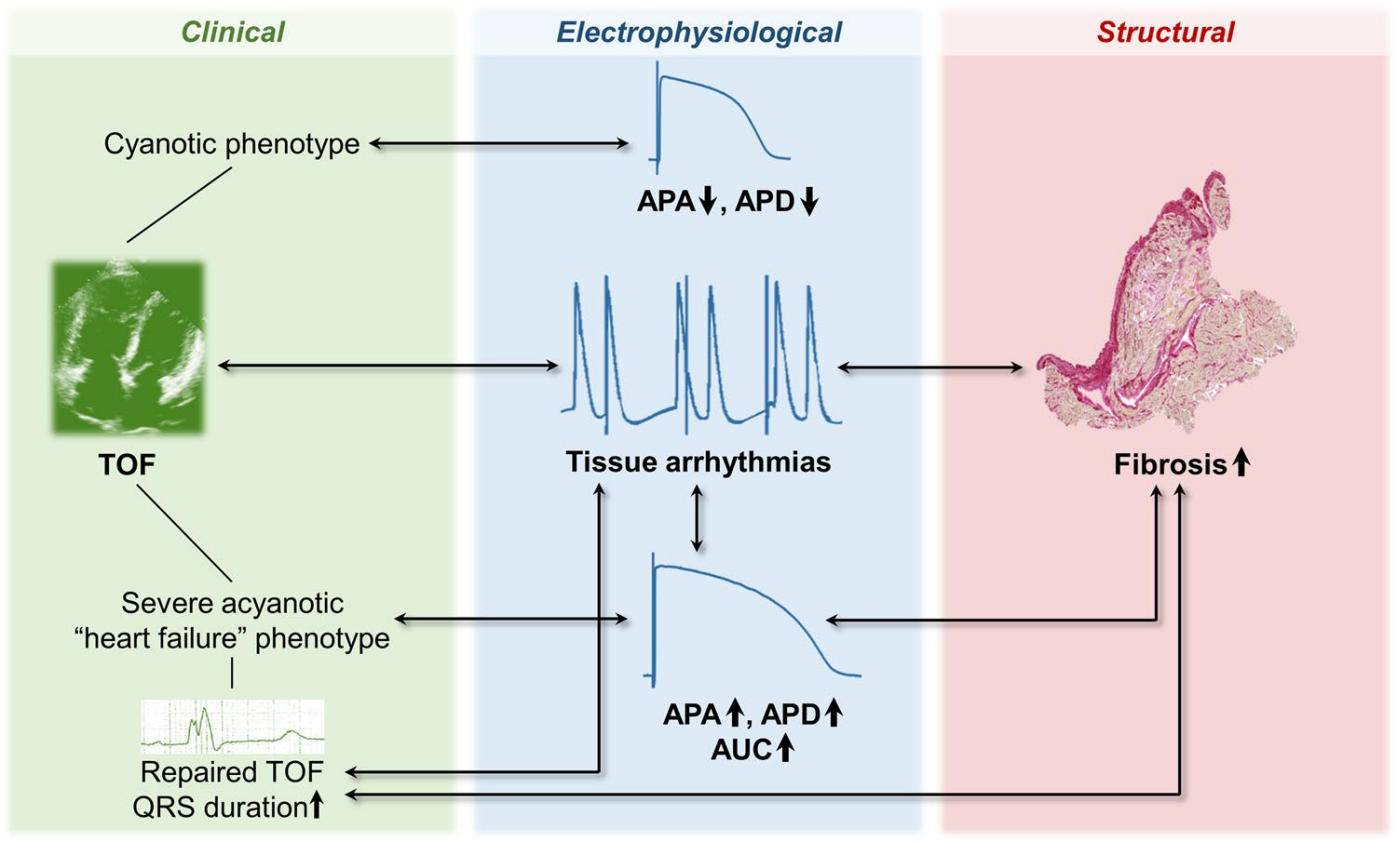

**Supplementary Information:**

The online version contains supplementary material available at 10.1007/s00392-023-02288-z.

## Introduction

Despite modern clinical care, patients with tetralogy of Fallot (TOF) remain at increased risk of ventricular arrhythmias and sudden cardiac death [1, 2]. Clinical electrophysiological investigations have identified the right ventricle (RV), and specifically the RV outflow tract (RVOT), as the place of origin of these arrhythmias [3, 4]. While post-operative surgical scars were previously assumed to be the main cause of ventricular arrhythmias, more recent data indicate that pro-arrhythmic perturbations in patients with TOF may originate from RV myocardium unaffected by surgical interventions [5, 6]. Similarly, subclinical electrocardiographic changes have been demonstrated in these patients both before and after surgical repair [7, 8], and studies of isolated RVOT cardiomyocytes from young children with unrepaired TOF have revealed frequent spontaneous early afterdepolarisations [9, 10]. In a porcine model of repaired TOF, action potential (AP) duration (APD) and conduction velocity were altered in RVOT wedges [11], consistent with the increased dispersion of repolarisation repeatedly observed in electrocardiograms of patients with TOF [7, 12]. However, electrophysiological remodelling of human RVOT cardiomyocytes from patients with TOF in their native multicellular environment and the extent of their pro-arrhythmic constitution remain unconfirmed. In addition, the links between clinical phenotype, indicators of disease severity, and cardiomyocyte electrophysiology have yet to be investigated.

The aim of this study was to characterise AP properties and myocardial arrhythmia susceptibility in patients with unrepaired and repaired TOF by intracellular membrane potential recording in RVOT myectomies, and to examine correlations between clinical phenotype and AP shape, including abnormal depolarisation or repolarisation. For comparison, we used RVOT biopsies from patients undergoing surgical closure of a large atrial septal defect (ASD). ASD is a much milder congenital heart defect than TOF, but nevertheless also affects the RV by volume overload and increases arrhythmic risk in adulthood [13–15].

TOF is typically considered a primarily structural rather than electrical disease. Histological and magnetic resonance imaging data have suggested that patients with TOF suffer from myocardial fibrosis (an excess accumulation of extracellular matrix) [16–21], and both focal/scar-associated and diffuse fibrosis seem to contribute to ventricular arrhythmias in repaired patients [22]. This line of thought is supported by data from porcine models of repaired TOF, in which RV fibrosis was a predictor of spontaneous arrhythmogenesis [11], whereas ventricular arrhythmias were not spontaneously present and could not be induced in the absence of RV fibrosis [23].

In patients with TOF, alterations of the mechanical environment of cardiomyocytes by both haemodynamic load and fibrosis may alter cardiomyocyte electrophysiology [24], potentially making patients more prone to ventricular arrhythmogenesis [24, 25]. We therefore hypothesised that electrophysiological remodelling of cardiomyocytes is more severe in the presence of pronounced RVOT fibrosis in patients with TOF. To address this hypothesis, the extent of fibrosis, AP properties, propensity for tissue arrhythmias, and clinical indicators potentially predisposing for severe remodelling were investigated.

## Materials and methods

### Tissue collection

RVOT samples were resected from patients with TOF and patients with ASD during repair operation or re-operation and collected, together with clinical patient data, by the CardioVascular BioBank (CVBB) of the University Heart Center Freiburg. The use of TOF and ASD tissue and data were approved by the Ethics Committee of the University of Freiburg (ethics vote number 393/16 for the CVBB, and 589/17 for this study). The study complies with the Declaration of Helsinki. Informed consent was obtained from all patients or their legal guardians.

### Intracellular membrane potential recording

For a detailed description of the methods, see Supplemental Material. In brief, live RVOT tissue was placed in a chamber continuously perfused with oxygenated, hypocalcaemic Krebs–Henseleit solution heated to physiological temperature. Under pacing with a frequency of 1 Hz, the calcium concentration in the solution was gradually raised to 1.8 mM. The tissue was impaled with a microelectrode filled with 3 M KCl and connected to a bridge amplifier, and the potential was recorded using a custom-made script in LabView software (National Instruments, Austin, TX, USA; script available from authors upon request). A minimum of 20 AP were recorded from at least three locations within a sample at stimulation frequencies of 0.5, 1, 2, 3, and 4 Hz each. Any spontaneous arrhythmias (‘tissue arrhythmias’) and other AP abnormalities were also recorded. Thereafter, arrhythmia provocation was performed by superfusion with a hypokalaemic Krebs–Henseleit solution containing BaCl_2_, and, in the case of stable conditions, with a normokalaemic Krebs–Henseleit solution containing isoprenaline. Any tissue arrhythmias or other AP abnormalities were recorded during drug provocation.

All recordings were visually examined for arrhythmias and abnormal AP shapes, and AP with artefacts, AP amplitude (APA; defined as potential difference, in mV, between the resting membrane potential [RMP] and the peak potential of the AP) below 75 mV, and those with insufficient separation of AP upstroke from the stimulation artefact were rejected. All AP without tissue arrhythmias and impaired APD shortening (see section ‘Pro-arrhythmic electrophysiological tissue abnormalities’) were then analysed with a custom Python script [26] to identify the AP shape properties RMP, APA, maximum upstroke velocity (d*V*/d*t*_max_), APD at 20%, 50%, and 90% repolarisation (APD_20_, APD_50_, APD_90_), and area under the curve at 90% repolarisation (AUC_90_). For the number of patients included in the analysis for each stimulation frequency, see Fig. S1.

### Histological fibrosis quantification

After AP measurements, RVOT tissue samples were histologically processed and analysed as previously described in [16] (see Supplemental Material). Briefly, a minimum of 30 alternate tissue sections per patient were batch-stained with picrosirius red. Automated fibrosis quantification in these images, yielding percent-fibrosis for each section, was performed with custom Python scripts as reported in [16]. In all but two patients the same sample was used for both electrophysiology and histology.

### Statistical analyses and clinical parameters

Statistical methods are described in detail in the Supplemental Material. In summary, we used a mixed linear effects model to evaluate associations of clinical parameters and tissue abnormalities with AP shape properties (dependent variables: RMP, APA, d*V*/d*t*_max_, APD_20_, APD_50_, APD_90_, AUC_90_). The following clinical and tissue parameters were defined as fixed effects: age, pre-operative clinical parameters (disease type, repair status, prolonged QRS duration [27, 28], cyanosis, echocardiographically measured pressure gradient between RV and pulmonary arteries [RV–PA gradient; categorised as mild, moderate, or severe], medication with beta blockers, pro-brain natriuretic peptide [proBNP] elevation [categorised as none or mild, and severe]), occurrence of pre- or post-operative clinical arrhythmias in vivo, electrophysiological tissue abnormalities (see section ‘Pro-arrhythmic electrophysiological tissue abnormalities’), as well as tissue pacing frequency. ‘Patient’ was defined as random effect.

The association of percent-fibrosis with AP properties at 1 Hz, clinical parameters, and electrophysiological tissue abnormalities was assessed using a mixed linear effects model with percent-fibrosis as the dependent variable, patient as random effect, and AP properties, clinical parameters, and electrophysiological tissue abnormalities (see above) as fixed effects.

The association of clinical parameters, electrophysiological tissue abnormalities, and fibrosis (as independent variables) with the occurrence of clinical arrhythmias and with the occurrence of tissue arrhythmias (as dependent variables) was evaluated by binomial logistic regressions. Comparison of AP properties between tissue with drug-induced arrhythmias and tissue with spontaneous arrhythmias was performed by two-sample *t*-test.

*p-*Values < 0.05 were considered as indicating statistical significance for all analyses. All values are given as mean ± standard error of the mean, unless indicated otherwise.

## Results

### Clinical patient characteristics

We performed AP measurements on RVOT samples from 25 patients with a mean age of 101 months (range 4 months to 56 years; Table S3). The underlying congenital heart defect was TOF in 22 and ASD in three patients. Six patients with TOF (median age 27 years) had previously undergone a repair operation (‘repaired patients’); all other patients (median age 10 months) had not previously received a repair operation (‘unrepaired patients’). All repaired patients demonstrated prolonged QRS durations in the pre-operative electrocardiogram, while all unrepaired patients had normal (age-adjusted) pre-operative QRS durations. Six patients experienced post-operative supraventricular or junctional arrhythmias, but none had ventricular arrhythmias or history of any kind of pre-operative tachyarrhythmia. For further clinical patient information, see Table 1 and Table S3.

**Table 1:** Clinical parameters and electrophysiological tissue abnormalities

*n* (%)	*N* = 25 (100)
Pre-operative clinical parameters, *n* (%)	
Sex	
Male	16 (64)
Female	9 (36)
Congenital heart defect	
Tetralogy of Fallot	22 (88)
Atrial septal defect	3 (12)
Repair status and QRS prolongation	
Unrepaired and normal QRS	19 (76)
Repaired and prolonged QRS	6 (24)
Cyanosis	
Acyanotic	16 (64)
Cyanotic	9 (36)
RV–PA pressure gradient	
Mild	12 (48)
Moderate	2 (8)
Severe	11 (44)
Beta blocker therapy	
Yes	9 (36)
No	16 (64)
ProBNP elevation	
None or mild	13 (52)
Severe	12 (48)
Post-operative SVT or junctional clinical arrhythmias^a^	
Yes	6 (24)
No	19 (76)
Electrophysiological tissue abnormalities, *n* (%)	
Tissue arrhythmias	
Yes	13 (52)
No	12 (12)
Impaired APD shortening	
Yes	6 (24)
No	19 (76)
APD alternans	
Yes	12 (48)
No	13 (52)

^a^All tachyarrhythmias occurred in the immediate post-operative phase; there was no manifestation of ventricular tachyarrhythmias or history of pre-operative tachyarrhythmias in these patients. *ProBNP* pro-brain natriuretic peptide, *RV–PA* right ventricle to pulmonary artery, *SVT* supraventricular tachycardia

### Pro-arrhythmic electrophysiological tissue abnormalities

**Fig. 1:**
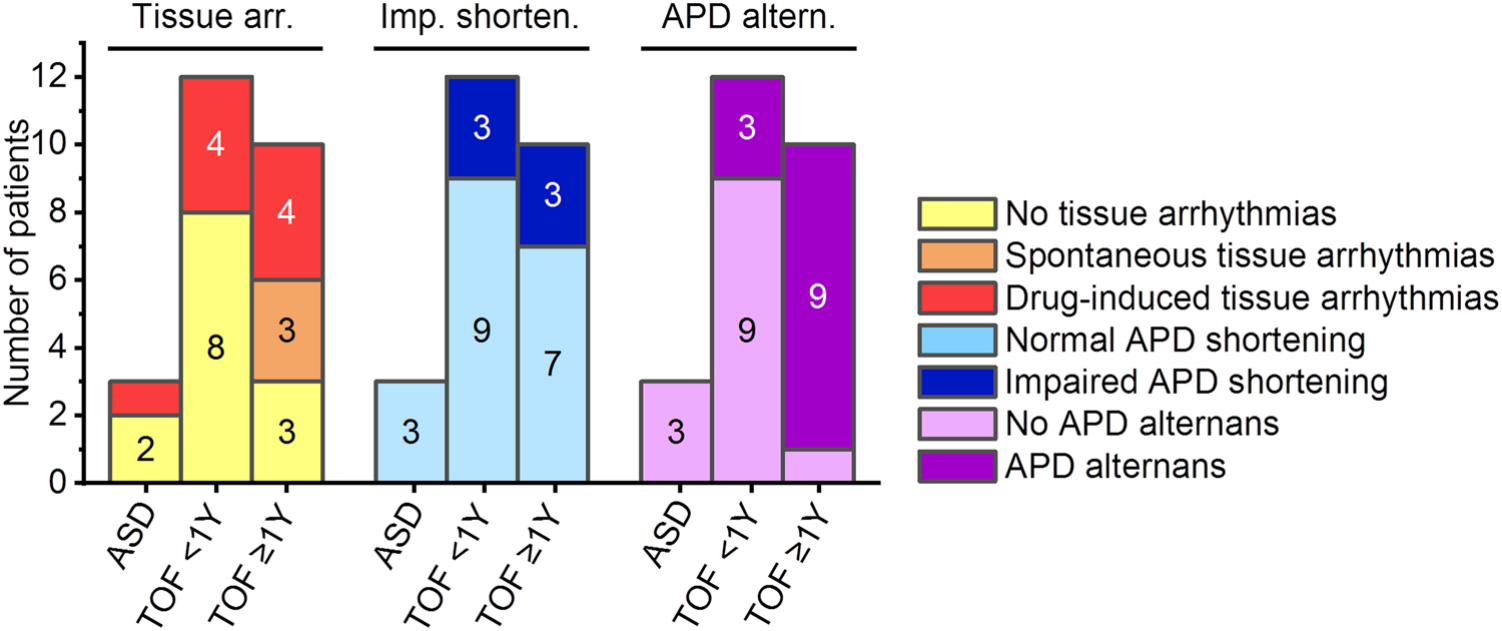
Occurrence of pro-arrhythmic electrophysiological tissue abnormalities in myocardium from patients with tetralogy of Fallot (TOF) and with atrial septal defect (ASD). *Altern.* alternans, *APD* action potential duration, *arr.* arrhythmias, *imp. shorten.* impaired APD shortening, *Y* years of age

During AP recording, 4 of 12 (33%) samples from young (< 1 year) and 7 of 10 (70%) samples from older (≥ 1 year) patients with TOF showed either spontaneous or drug-induced arrhythmias. In the ASD group, 1 of 3 (33%) samples showed a drug-induced arrhythmia (Fig. 1, Table 1). There was no significant difference between tissue with drug-induced and tissue with spontaneous arrhythmias in RMP, d*V*/d*t*_max_, APD_20_, APD_50_, APD_90_, and AUC_90_ of the non-arrhythmic AP over all pacing frequencies assessed (two-sample *t*-test, *p* > 0.05 for all). APA was slightly larger in tissue with drug-induced arrhythmias compared to tissue with spontaneous arrhythmias (two-sample *t*-test, *p* = 0.023).

**Fig. 2:**
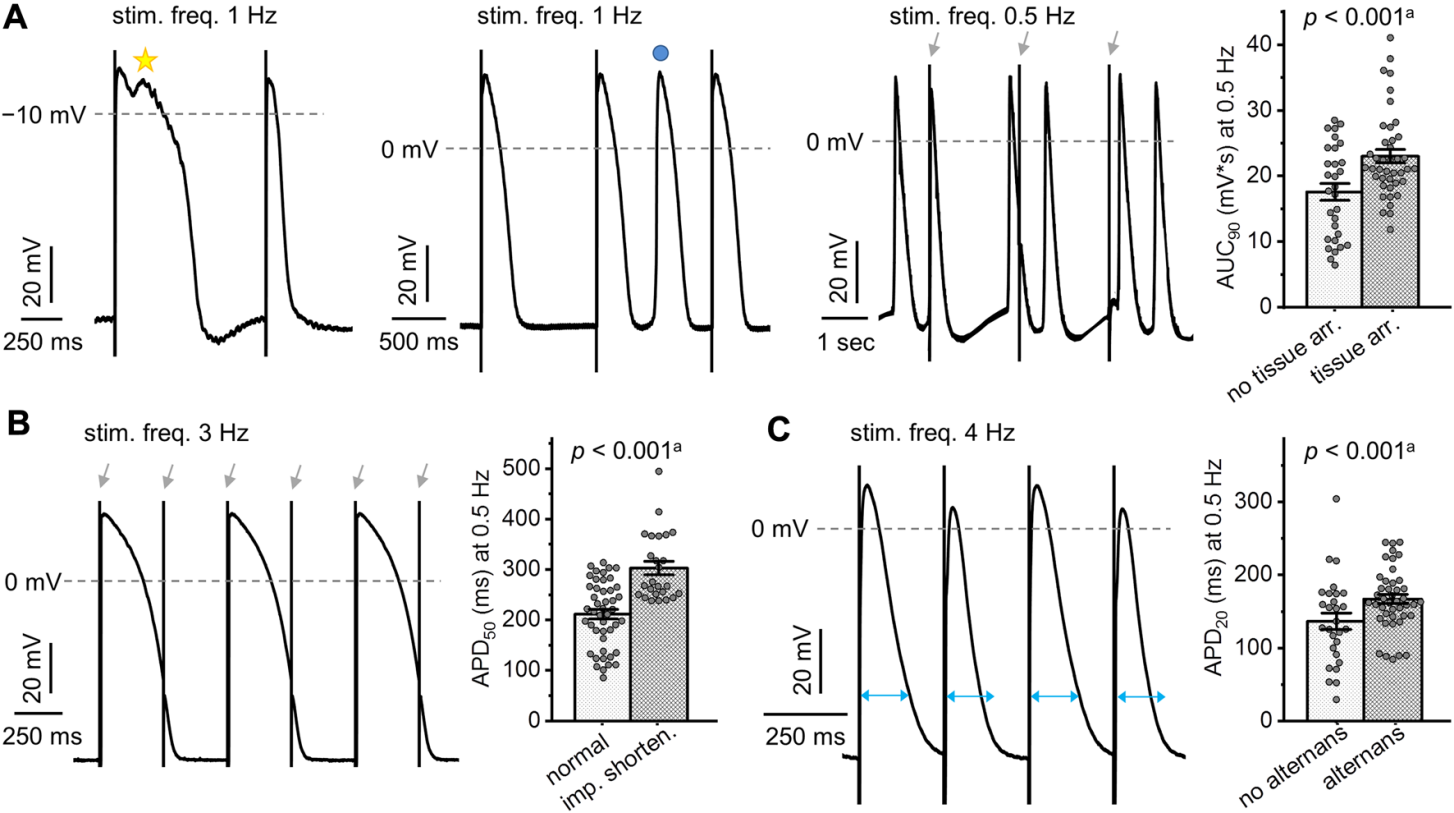
Pro-arrhythmic electrophysiological tissue abnormalities in myocardial samples and their association with action potential (AP) shape. **A** Example traces of tissue arrhythmias: early afterdepolarisations (left, indicated by yellow star), extrasystoles (middle, indicated by blue circle), and stimulation-independent spontaneous depolarisations (right, grey arrows indicate stimulation timing). **B** Example of impaired action potential duration (APD) shortening at stimulation frequency of 3 Hz (grey arrows indicate stimulation timing). **C** Example APD alternans with blue arrows indicating alternating APD. ^a^Bar graphs indicate one data point per recording location; *p*-Values represent association over all frequencies for a given AP parameter and occurrence of electrophysiological tissue abnormality in mixed linear effects model. *arr.* arrhythmias, *AUC*_*90*_ area under the curve at 90% repolarisation, *imp. shorten.* impaired APD shortening, *stim. freq.* stimulation frequency

Three types of tissue arrhythmias were observed: (i) early afterdepolarisations, (ii) extrasystoles between triggered AP, and (iii) stimulation-independent, spontaneous depolarisations in the form of couplets (Fig. 2A). Other pro-arrhythmic electrophysiological tissue abnormalities included impaired APD shortening with increasing stimulation frequency, resulting in a failure to follow increased stimulation rates of 2, 3, and/or 4 Hz (Fig. 2B), and APD alternans (Fig. 2C), which were both frequently observed in TOF but not in ASD tissue (Fig. 1).

### Association of AP shape with stimulation frequency, clinical parameters, and electrophysiological tissue abnormalities

An overview of the readouts from the mixed linear effects model analysis of the AP data, including estimates and *p*-values, is presented in Table 2.

**Table 2:** Mixed linear effects model results of electrophysiological data

	RMP		APA		*d*V/*d*t_max_		APD_20_		APD_50_		APD_90_		AUC_90_	
	Est. (mV)	*p*	Est. (mV)	*p*	Est. (V/s)	*p*	Est. (ms)	*p*	Est. (ms)	*p*	Est. (ms)	*p*	Est. (mV*s)	*p*
Intercept	** − 73.40**	**0.000**	**82.87**	**0.000**	**124.16**	**0.000**	**112.16**	**0.000**	**157.43**	**0.000**	**217.62**	**0.000**	**11.86**	**0.000**
Stim. frequency	0.38	0.272	** − 1.38**	**0.000**	**-15.85**	**0.000**	** − 22.78**	**0.000**	** − 37.32**	**0.000**	** − 41.46**	**0.000**	** − 3.22**	**0.000**
Disease type	− 3.98	0.085	5.06	0.236	17.89	0.361	− 5.02	0.629	29.16	0.060	37.08	0.310	**3.49**	**0.014**
Repair status	0.11	0.968	− 0.57	0.916	**76.90**	**0.002**	** − 59.93**	**0.000**	− 31.86	0.094	12.30	0.792	− 2.74	0.116
Age	0.10	0.207	0.01	0.963	**-1.59**	**0.022**	**1.50**	**0.000**	**1.56**	**0.004**	0.98	0.365	**0.16**	**0.001**
Cyanosis	1.81	0.099	** − 4.17**	**0.047**	** − 31.32**	**0.009**	2.00	0.688	**21.05**	**0.005**	**41.21**	**0.023**	1.15	0.093
Mod. RV-PA grad	1.35	0.528	**13.38**	**0.001**	**36.16**	**0.044**	** − 21.60**	**0.027**	12.26	0.397	41.16	0.247	**3.51**	**0.008**
Sev. RV-PA grad	0.48	0.799	5.36	0.135	2.27	0.887	− 10.88	0.201	20.47	0.107	45.84	0.138	**2.73**	**0.020**
ProBNP elevation	− 0.72	0.497	3.78	0.062	11.88	0.178	**23.42**	**0.000**	**26.36**	**0.000**	16.37	0.351	**2.80**	**0.000**
Beta blockers	− 1.63	0.130	**6.05**	**0.006**	**29.94**	**0.001**	8.87	0.066	**25.18**	**0.001**	31.39	0.097	**3.13**	**0.000**
Clinical arrhythm	0.30	0.774	− 1.81	0.396	**27.32**	**0.001**	** − 12.22**	**0.010**	** − 29.27**	**0.000**	** − 56.37**	**0.002**	** − 2.80**	**0.000**
Tissue arrhythm	0.78	0.477	**5.11**	**0.013**	15.28	0.087	**27.92**	**0.000**	**43.80**	**0.000**	**41.41**	**0.019**	**4.52**	**0.000**
Imp. APD short	− 0.59	0.556	2.42	0.231	**-34.58**	**0.000**	**52.68**	**0.000**	**93.51**	**0.000**	**127.82**	**0.000**	**8.17**	**0.000**
APD alternans	1.05	0.321	2.35	0.258	10.71	0.218	**28.82**	**0.000**	6.67	0.351	− 24.15	0.178	0.42	0.529

For details regarding statistical analysis, see text. Statistically significant results are presented in bold type, non-significant results in normal type

*APA* action potential amplitude*, APD* action potential duration*, arrhythm.* arrhythmia, *AUC* area under the curve, *est.* estimate, *grad.* gradient, *imp. APD short.* impaired APD shortening with increasing stimulation frequency, *mod.* moderate, *RV-PA grad.* right ventricle to pulmonary artery pressure gradient, *sev.* severe, *stim.* stimulation

**Fig. 3:**
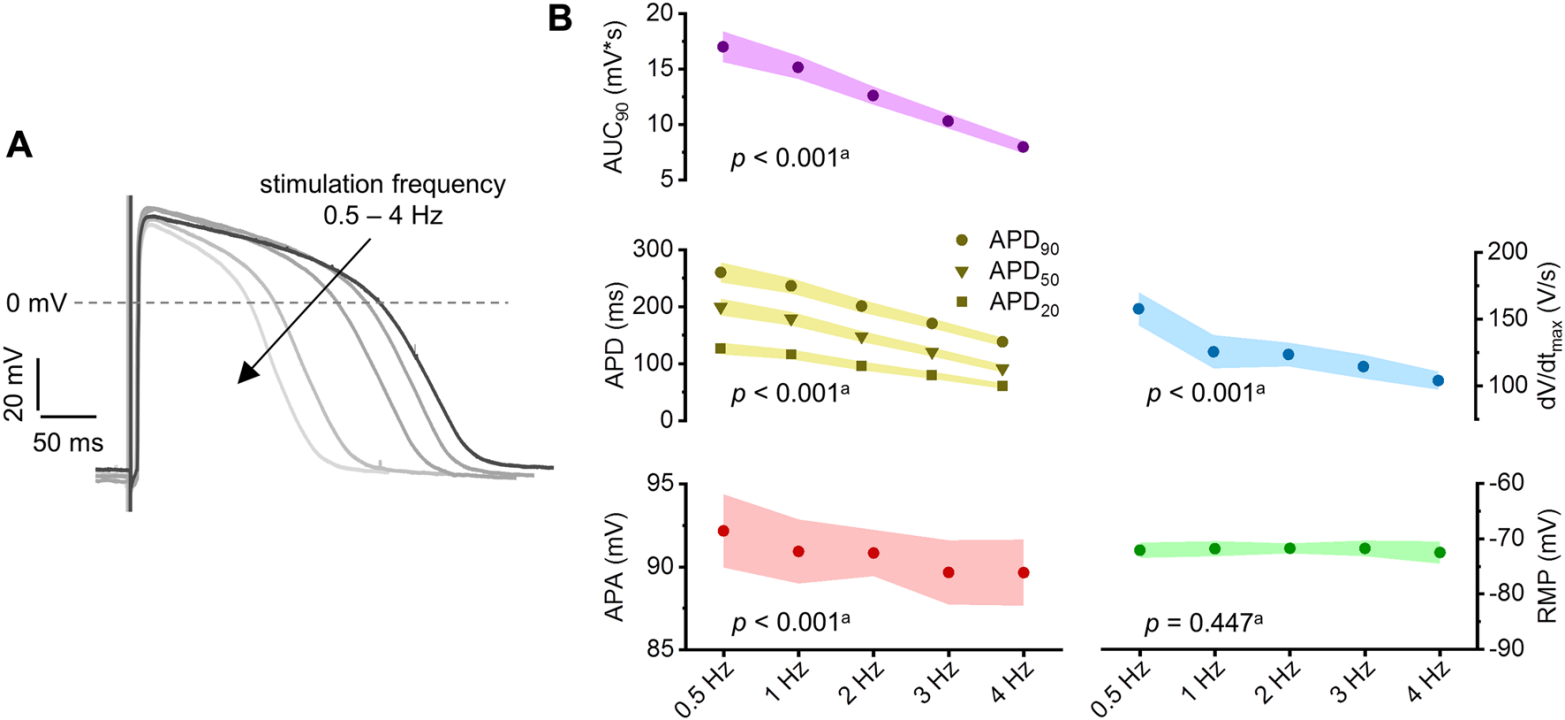
Frequency response of action potential (AP) properties. **A** Representative AP traces of a sample from a 5-month-old unrepaired tetralogy of Fallot patient showing decreasing AP duration (APD) with increasing stimulation frequency. **B** AP parameters at different stimulation frequencies averaged across patients (excluding patients with impaired APD shortening). ^a^*p*-Values shown for association of given AP parameter with stimulation frequency in mixed linear effects model. *APA* action potential amplitude, *AUC* area under the curve, *dV/dt*_*max*_ maximum upstroke velocity, *RMP* resting membrane potential

Despite six patients with TOF showing impaired APD shortening in a subset of recording locations, the AP properties APA, d*V*/d*t*_max_, APD, and AUC_90_ decreased significantly with increasing stimulation frequency in the averages across all recording locations (Fig. 3, Table 2, Table S1, Table S2). There was no significant effect of stimulation frequency on RMP.

**Fig. 4:**
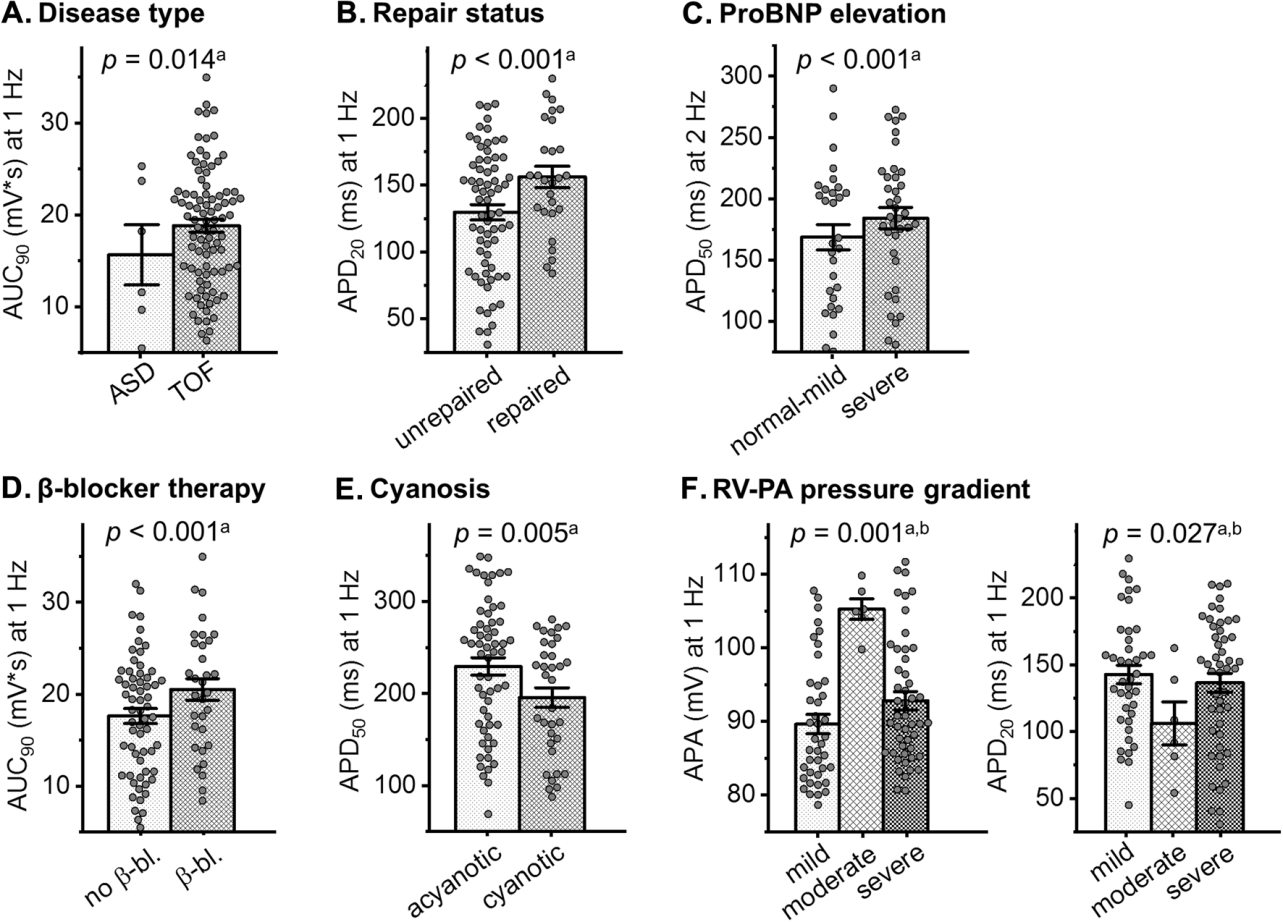
Association of clinical parameters with action potential (AP) properties. **A–F** Example graphs showing association of clinical parameters with AP parameters. ^a^Bar graphs indicate one data point per recording location; *p*-values shown for given association over all frequencies in mixed linear effects model. ^b^*p*-Values for given AP parameter in moderate versus mild right-ventricle-to-pulmonary-artery (RV–PA) pressure gradient elevation over all frequencies. *APA* action potential amplitude, *APD* action potential duration, *AUC* area under the curve, *β-bl.* beta blocker

Evaluating the association of clinical parameters with AP shape demonstrated a statistically significant positive association of the disease type TOF with larger AUC_90_, of repaired status with faster d*V*/d*t*_max_ and longer APD_20_, of severe proBNP elevation with longer APD_20_, APD_50_, and larger AUC_90_, and of beta blocker treatment with larger APA, faster d*V*/d*t*_max_, longer APD_50_, and larger AUC_90_ (Fig. 4A–D, Table 2). In contrast, there was a statistically significant negative association of cyanosis with APA, d*V*/d*t*_max_, APD_50_, and APD_90_ (Fig. 4E, Table 2), and of clinical arrhythmias with APD_20_, APD_50_, APD_90_, and AUC_90_. The different grades of RV–PA pressure gradient elevation showed differential effects, with significantly larger APA and faster d*V*/d*t*_max_ in moderate RV–PA gradient elevation compared to mild and severe RV–PA gradient elevation, and significantly shorter APD_20_ and smaller AUC_90_ in moderate RV–PA gradient elevation compared to mild RV–PA gradient elevation (Fig. 4F, Table 2). Finally, age was significantly associated with d*V*/d*t*_max_, APD_20_, APD_50_, and AUC_90_; however, when taking into account the small estimates (Table 2) and the graphical presentation in Fig. S2, interpretation and biological relevance appear limited.

Evaluating the association of pro-arrhythmic tissue abnormalities with AP parameters demonstrated a significant association of tissue arrhythmias with larger APA, longer APD_20_, APD_50_, and APD_90_, and larger AUC_90_, of impaired APD shortening with slower d*V*/d*t*_max_, longer APD_20_, APD_50_, and APD_90_, and larger AUC_90_, and of APD alternans with longer APD_20_ (Fig. 2, Table 2).

No other results from the above mixed linear effects analysis showed statistical significance. As revealed by adjusted *R*^2^, the variability covered by the model was 5% for RMP, 47% for APA, 28% for d*V*/d*t*_max_, 64% for APD_20_, 67% for APD_50_, 77% for APD_90_, and 66% for AUC_90_.

Binomial logistic regression demonstrated a significant positive interaction of repaired status/QRS prolongation with tissue arrhythmias (*t* =  − 2.0, *p* = 0.045), while there was no significant association of clinical arrhythmias with any clinical parameters or electrophysiological tissue abnormalities.

### Relation of myocardial fibrosis to clinical parameters and tissue electrophysiology

**Fig. 5:**
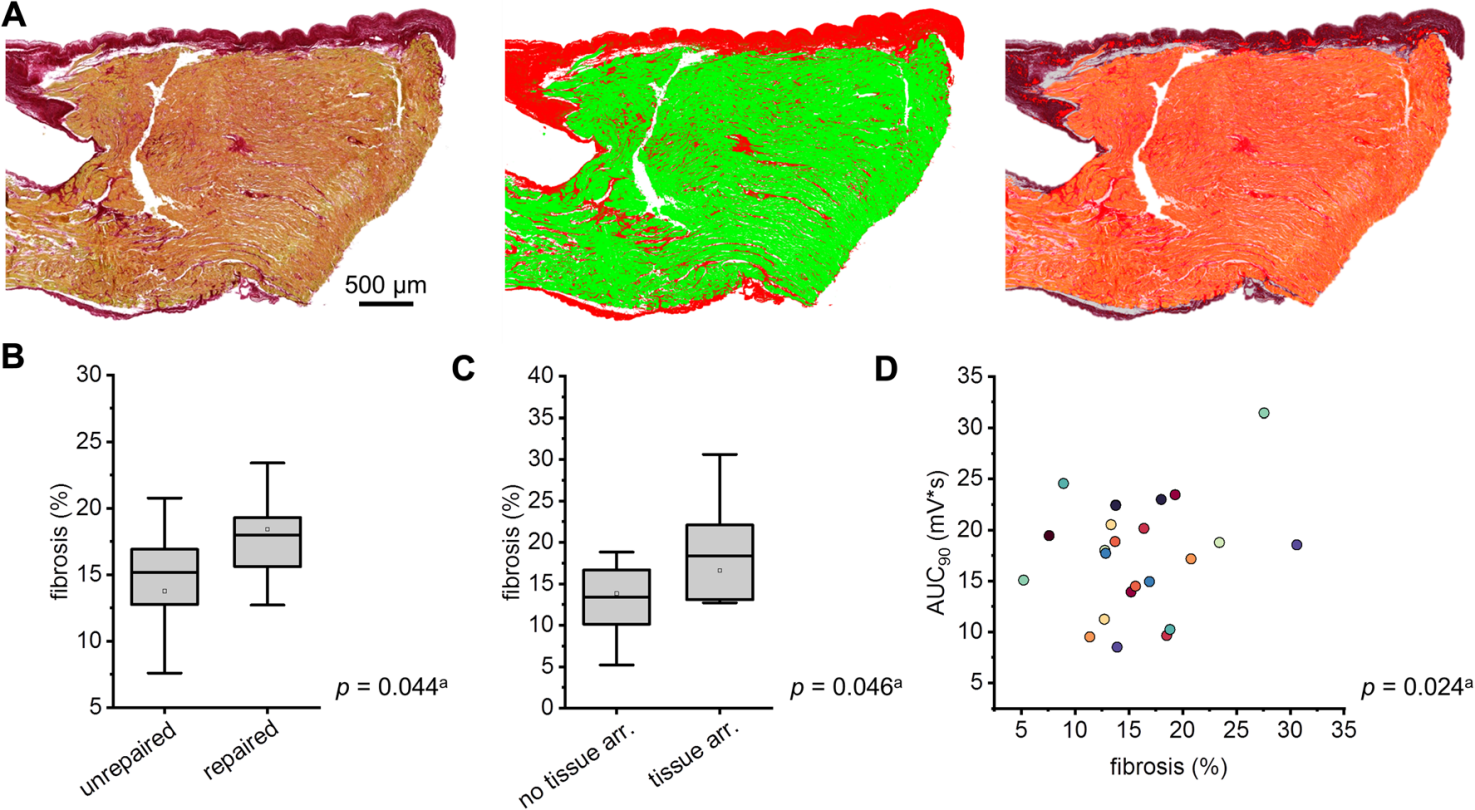
Association of tissue fibrosis with clinical parameters and action potential (AP) properties. **A** Example image of a right ventricular sample section of a repaired tetralogy of Fallot patient, stained with picrosirius red (red: collagen, yellow: myocardium) (left); the corresponding segmentation into collagen (red) and myocardium (green) (middle); and the area analysed for percent-fibrosis (orange) after exclusion of background and tissue gaps (white), thickened endocardium/other non-myocardial collagen-containing structures (dark red) (analysis script reported in [16]). **B–C** Association of fibrosis with repair status and tissue arrhythmia (arr.). **D** Association of area under the curve at 90% repolarisation (AUC_90_) with fibrosis; each data point indicates one patient sample. ^a^*p*-Values shown for given association in mixed linear effects model

Mean percent-fibrosis for all patients, patients with TOF, and patients with ASD was 15.9 ± 1.2%, 15.9 ± 1.3%, and 15.4 ± 1.6%, respectively. An example histological stain is depicted in Fig. 5A, and individual patient values for percent-fibrosis are shown in Table S3. Evaluation of the association of clinical parameters, electrophysiological tissue abnormalities, and AP shape properties with fibrosis revealed a positive association of repaired TOF status/QRS prolongation, tissue arrhythmias, and larger AUC_90_ with increased percent-fibrosis (estimate [est.] 20.4%, *p* = 0.044; est. − 7.0%, *p* = 0.046; and est. 11.1%, *p* = 0.024, respectively) (Fig. 5B–D). Age, RMP, and APA were also significantly associated with percent-fibrosis, but only with small estimates of − 0.7% (*p* = 0.010), 0.6% (*p* = 0.023), and − 1.6% (*p* = 0.030), respectively, suggesting little to no biological relevance (Fig. S2). No other results from this analysis were statistically significant. Notably, there was no significant association between cyanosis or RV–PA pressure gradient and percent-fibrosis (est. − 6.9%, *p* = 0.056 for cyanosis; est. 9.2%, *p* = 0.206 for moderate RV–PA gradient; est. 6.4%, *p* = 0.221 for severe RV–PA gradient).

## Discussion

Our data confirms pronounced arrhythmogenic potential in myocardium from patients with TOF that is already present at a young age. In addition, tissue arrhythmias were associated with repair status, QRS duration, and fibrosis. Cyanotic patients had shorter APD and smaller APA, while more severely diseased, acyanotic patients with a heart-failure-like phenotype, and those with increased myocardial fibrosis and tissue arrhythmias demonstrated longer APD and larger AUC and/or larger APA (see Central Illustration/Graphical Abstract).

The detection of pro-arrhythmic changes in tissue electrophysiology not only in RV myocardium of adult patients with repaired TOF and prolonged QRS duration, but also in young patients before repair operation, unequivocally indicates an early, subclinical onset of pro-arrhythmic remodelling regardless of surgical intervention. Therefore, we confirmed that arrhythmogenesis in TOF is not limited to patients with surgical scars in the myocardium after myotomy in the RVOT with or without a ventriculotomy and insertion of a transannular patch, but may also be caused by other myocardial remodelling in the RVOT unrelated to surgery, thereby supporting and extending the findings of a previous small-scale study describing early afterdepolarisations in isolated cardiomyocytes from children with unrepaired TOF [10]. Nevertheless, tissue arrhythmias were more likely to occur in the repaired group, which reflects the well-known risk of clinical arrhythmia affecting patients with repaired TOF and prolonged QRS duration [29, 30]. Our results call for closer investigation of the underlying pro-arrhythmic mechanisms in the myocardium and highlight the importance of clinically monitoring not just adult patients, but younger patients too.

More severely diseased, acyanotic patients with a heart-/RV-failure-like phenotype and clinically longer-standing disease (i.e. repaired TOF, acyanosis, severe proBNP elevation, necessity of beta blocker treatment, moderate RV-PA gradient) and more extensively remodelled tissue (i.e. more tissue electrophysiological abnormalities, fibrosis) showed longer APD and/or larger AUC. As the clinical background of these patients includes, above all, an enhanced RV load that may be accompanied by (subclinical) RV functional impairment or failure, patients with TOF may demonstrate electrophysiological remodelling similar to adult patients without congenital heart disease, who have shown longer APD and QTc in right and left heart failure [31–35]. Clinically, it is also well known that heart failure in general, and RV dilation and failure in repaired TOF in particular, are risk factors for ventricular arrhythmias and sudden cardiac death [36–39]. Moreover, (inducible) ventricular tachycardia in the RVOT has been associated with prolonged APD in animal models [40]. Our results bring together these clinical and experimental findings, as the occurrence of tissue arrhythmias in patients with TOF was greater in myocardial samples with longer APD and larger AUC. Thus, our data raise the question of whether electrophysiological changes in patients with congenital heart disease are an inherent result of the disease itself, or whether they may be attributed to secondary events such as haemodynamic abnormalities of different origins but with similar effects on the myocardium. Further investigation of the patient population with congenital heart defects is urgently needed to answer this question.

In contrast to this heart-failure-associated, pro-arrhythmic AP phenotype, cyanotic patients demonstrated smaller APA and shorter APD. This is consistent with animal models investigating the effect of short-term hypoxia on AP shape [41], but contrasts with a mouse model of cyanotic disease exhibiting slower conduction velocity, longer QTc, and gene expression alterations of cardiac ion channels in chronic pre- and post-natal hypoxia [42]. Cyanosis, therefore, appears to alter the electrophysiology differently than other indicators of disease severity in TOF, highlighting that structural and haemodynamic differences within a common underlying congenital heart defect may lead to distinct electrophysiological remodelling, possibly with different arrhythmic risk profiles.

Unexpectedly, the extent of fibrosis was not significantly different between TOF and ASD samples, despite ASD being a much less severe disease, at least in early childhood. This fibrosis could possibly be attributed to the extent of RV volume overload, which was not insignificant in the ASD patients with large defect size included here, and would suggest that ASD may also already affect RV myocardium at a young age, contrary to the current clinical perception.

We found increased fibrosis in repaired patients when compared to unrepaired patients, which is consistent with progressive fibrotic remodelling due to pulmonary insufficiency and concomitant volume overload in the RV, as shown previously [11, 17, 43]. In addition, our results demonstrating increased myocardial fibrosis both in repaired patients and in tissue with arrhythmias are in line with clinical findings that have related ventricular arrhythmogenesis to diffuse and focal fibrosis in cardiac magnetic resonance imaging of patients with repaired TOF [22]. Possibly, if both the pro-arrhythmic electrical abnormalities and the previously described effects of surgical scars in repaired TOF were dependent on fibrosis, these two arrhythmia mechanisms may be interrelated and therefore enhance the risk of ventricular arrhythmias in patients with repaired TOF. However, the question of whether there is a causal link between fibrosis and AP remodelling in patients with congenital heart disease, via electromechanical interactions for example, cannot be determined from our data and warrants further investigation.

### Study limitations

The interpretability of some of our results was limited by the high inter-individual variability of patients with congenital heart disease. An additional limitation was the small number of patients in the comparator group (i.e. patients with ASD). Despite the significant association of age with AP shape properties and the extent of fibrosis, for example, the small estimates of < 1.6 ms for APD_20_ and APD_50_ and of 0.16 mV*s for AUC_90_ serve as a reminder that ‘significance’ is not synonymous with ‘relevance’ (Table 2, Fig. S2). Clinically, there is a well-known effect of age on arrhythmogenesis in repaired TOF [44]; unfortunately, however, the influence of age on AP remodelling remains unclear in this study.

The absence of pre- and peri-operative clinical ventricular arrhythmias in our patient group only allows for indirect assumptions regarding the links between ex situ tissue findings and the risk of clinically relevant rhythm disturbances. In the study population, clinical tachyarrhythmias were of either supraventricular or junctional origin, manifesting exclusively during the immediate post-operative phase. Therefore, they are presumably caused by other factors (such as cardiopulmonary bypass, electrolyte imbalances, or catecholamine infusion) rather than the underlying congenital heart disease and associated electrophysiological remodelling.

## Conclusions and outlook

Frequent pro-arrhythmic activity that already occurs at a young age in TOF RVOT myocardium may indicate early electrophysiological remodelling or an underlying arrhythmic predisposition, thereby confirming the presence of arrhythmogenic mechanisms unrelated to and in addition to the pro-arrhythmic effects of previously induced surgical scars. We identified two different forms of AP remodelling, highlighting differential electrophysiological responses in the study cohort despite common underlying congenital heart defects. As all phases of the AP were affected to some extent, it may be illuminating to examine ion currents in cardiomyocytes, as well as electrical conduction in these patients in future work. Finally, our results highlight the potential diagnostic value of monitoring myocardial fibrosis, once the causal links to electrophysiological function have been investigated further.

## Clinical perspectives

As we confirmed a potential link between fibrotic remodelling and pro-arrhythmic propensity in TOF myocardium, the rapidly improving clinical-imaging-based detection of structural alterations could aid the prediction of pro-arrhythmic risk in these patients once larger-scale studies have identified underlying mechanisms and their relation to clinical outcomes. In addition, long-term follow-up of the study patients for arrhythmias and heart failure symptoms, and linking these to myocardial findings, may provide additional information that is useful for clinical identification of higher-risk patients.

### Supplementary Information

Below is the link to the electronic supplementary material.

Supplementary file1 (DOCX 361 KB)

## Data Availability

Raw data are available from the authors upon request.

## Abbreviations

AP: Action potential
APA: Action potential amplitude
APD: Action potential duration
ASD: Atrial septal defect
AUC: Area under the curve
d*V*/d*t*_max_: Maximum upstroke velocity
est.: Estimate
proBNP: Pro-brain natriuretic peptide
RMP: Resting membrane potential
RV: Right ventricle
RVOT: Right ventricular outflow tract
RV–PA gradient: Right-ventricle-to-pulmonary-artery pressure gradient
TOF: Tetralogy of Fallot
