# Supplementary material for: Disease severity, arrhythmogenesis, and fibrosis are related to longer action potentials in tetralogy of Fallot
Source: Clin Res Cardiol. 2023 Sep 19;113(5):716–27. doi: 10.1007/s00392-023-02288-z (PMC11026253; doi:10.1007/s00392-023-02288-z)
Supplement: Supplementary file 1 — Supplementary file1 (DOCX 361 KB) [file 392_2023_2288_MOESM1_ESM.docx]

Supplemental Material

to the manuscript

“Disease severity, arrhythmogenesis, and fibrosis are related to longer action potentials

in tetralogy of Fallot”

by H.E. Fürniss *et al.*

# Supplemental Methods

## Intracellular membrane potential recording

After excision, live RVOT tissue was promptly placed in a calcium-free glucose-electrolyte solution (NaCl, 150 mM; KCl, 5.4 mM; KH_2_PO_4_, 2 mM; MgSO_4_, 2 mM; taurine, 11 mM; 3-(n-morpholino)propanesulfonic acid, 10 mM; 2,3-butanedione monoxime, 1.9 mM; glucose, 1.4 mM; pH = 7.4). Tissue was then transported to the electrophysiology laboratory where the tissue was pinned, with the endocardial side of the tissue facing upward, to the bottom of a custom-made, water-jacketed chamber with a volume of 8.5 mL. The chamber was continuously perfused at 46 mL/min with hypocalcaemic Krebs–Henseleit solution (NaCl, 118 mM; KCl, 4.7 mM; CaCl_2_, 0.2 mM; MgSO_4_, 0.91 mM; glucose, 5.55 mM; KH_2_PO_4_, 1.2 mM; NaHCO_3_, 20.0 mM; Na pyruvate, 2.0 mM) oxygenated with carbogen (95% O_2_, 5% CO_2_) and heated to 36.8 ± 0.2 °C as measured in the bath (pH = 7.4). For initial pacing of the preparation at a frequency of 1 Hz, we electrically stimulated the tissue with a point electrode (SNE-100, World Precision Instruments, Sarasota, FL, USA) controlled by a MyoPacer (IonOptix, Westwood, MA, USA). The tissue was left to adapt for 10 minutes, before starting ‘calcium recovery’, *i.e.* gradually raising the calcium concentration to 1.8 mM.

Microelectrodes were produced from glass capillaries (Clark borosilicate standard wall with filament, OD 1.00, ID 0.58, length 100 mm; Warner Instruments, Holliston, MA, USA) with a Sutter P-97 Flaming/Brown Micropipette Puller (Sutter Instrument, Novato, CA, USA), filled with 3 M KCl, and connected to a bridge amplifier (BA-01X, NPI Electronic, Tamm, Germany). Microelectrode resistance was 5 to 20 MΩ. An Ag-AgCl reference electrode was placed in the bath. The tissue was impaled with the microelectrode using a micromanipulator (Sensapex, Oulu, Finland) and the potential was recorded (sampling rate 50 kHz) using a custom-made script created using LabView software (National Instruments, Austin, TX, USA; script available from authors upon request). Whether impalement of a cardiomyocyte was successful or not was judged based on the resting membrane potential (RMP), the AP amplitude (APA; defined as potential difference, in mV, between the RMP and the peak potential of the AP), and the APD at 90% repolarisation (APD_90_), which were measured live by the software during membrane potential recording. A minimum of 20 AP were recorded from at least three locations within a sample at stimulation frequencies of 0.5, 1, 2, 3, and 4 Hz each. After changes of stimulation frequency, AP were left to stabilise for 20 to 30 seconds before sampling data for analyses. Any spontaneous arrhythmias (termed ‘tissue arrhythmias’, as opposed to ‘clinical arrhythmias’ that refer to arrhythmias seen in patients in vivo) or other AP abnormalities were also recorded. Following these measurements, arrhythmia provocation was performed by superfusion with a hypokalaemic (2.0 mM KCl), normocalcaemic (1.8 mM CaCl_2_) Krebs–Henseleit solution containing 120 µM BaCl_2_ for 15 minutes, followed by a 15-minute washout period. In the case of stable conditions, perfusion with 1 µM isoprenaline-containing, normokalaemic (4.7 mM KCl), normocalcaemic (1.8 mM CaCl_2_) Krebs–Henseleit solution was performed. Any arrhythmic AP (*i.e.* tissue arrhythmias) were recorded during drug provocation.

## Histological fibrosis quantification

After AP measurements, RVOT tissue samples were histologically processed and analyzed as previously described in Wülfers et al.^1^ (corresponds to reference 16 of the manuscript). Tissue was fixed in 4% paraformaldehyde, dehydrated, and embedded in wax before cutting into 10 µm-thick sections. A minimum of 30 alternate sections per tissue sample and patient were batch-stained with picrosirius red (ST5010 Autostainer XL, Leica, Wetzlar, Germany) to distinguish collagen (red) from cytoplasm and nuclei (yellow). All stained sections were automatically cover-slipped (CV5030 Fully Automated Glass Coverslipper, Leica, Wetzlar, Germany) and digitised (Axio Scan.Z1, Carl ZEISS AG, Oberkochen, Germany). Automated fibrosis quantification in these images, performed with custom Python scripts as reported in Wülfers et al.^1^ (reference 16 of the manuscript; source code available online), was based on detection of picrosirius red-stained intramyocardial collagen, and yielded percent-fibrosis for each section after exclusion of non-myocardial and/or collagen-dominated structures (especially thickened endocardium).

## Statistical analyses and clinical parameters

After exclusion of low-quality AP as described above, we analysed AP recordings from 1 to 7 locations per sample (average 3.8 per patient). To account for high intra-individual variability, we averaged the AP parameters per recording location (rather than per patient) for statistical analysis. Two RMP data points (values: −101.7 mV and −99.1 mV) were classified as outliers (outliers were defined as values lying below the first quartile − 3 × interquartile range or above the third quartile + 3 × interquartile range of the respective parameter) and excluded from further analysis. We used a mixed linear effects model implemented in MATLAB (MathWorks, Natick, MA, USA) to evaluate the possible associations of clinical parameters and tissue abnormalities with AP shape properties (dependent variables: RMP, APA, dV/dt_max_, APD_20_, APD_50_, APD_90_, AUC_90_). The following clinical and tissue parameters were defined as fixed effects: age (in years), disease type (TOF, ASD), pre-operative repair status (repaired, unrepaired), pre-operative presence of prolonged QRS duration (reference values from Davignon et al.^2^ for paediatric patients and Surawicz et al.^3^ for adult patients [correspond to references 27 and 28 of the manuscript, respectively]), pre-operative presence of cyanosis (defined as oxygen saturation < 90%, or < 93% with hypercyanotic spells), pre-operative echocardiographically measured pressure gradient between RV and pulmonary arteries (RV–PA pressure gradient, divided into three categories: mild [< 40 mmHg], moderate [40 – 60 mmHg], severe [> 60 mmHg]), pre-operative medication with beta blockers, pre-operative degree of pro-brain natriuretic peptide (proBNP) elevation (none or mild, < 400 ng/mL; severe, ≥ 400 ng/mL), occurrence of pre- or post-operative clinical arrhythmias, as well as electrophysiological tissue abnormalities observed during AP recording, namely occurrence of tissue arrhythmias, occurrence of APD alternans, and occurrence of impaired APD shortening upon increased stimulation frequency, and pacing frequency with 1:1 capture (tested values were 0.5, 1, 2, 3, and 4 Hz). As there were no relevant significant differences in AP parameters between tissue with spontaneous arrhythmias and tissue with drug-induced arrhythmias (see section ‘Pro-arrhythmic electrophysiological tissue abnormalities’ of the manuscript), the occurrences of both tissue arrhythmia types were considered as one group for the mixed linear effects analysis. The grouping variable for the AP at the different recording locations was the individual patient (*N* = 25), therefore ‘patient’ was defined as the random effect.

For histological analysis, one TOF patient sample had to be excluded due to poor staining quality. We analysed the extent of fibrosis in an average of 66 sections per patient (range 29 to 138 sections per patient; *N* = 21 TOF patients and *N* = 3 ASD patients; a total of 1,591 sections were assessed). Percent-fibrosis measured in histological sections was averaged by patient (for rationale, see Wülfers et al.^1^ [reference 16 of the main manuscript]). The association of percent-fibrosis with AP properties at 1 Hz (recording locations averaged by patient), clinical parameters, and electrophysiological tissue abnormalities was assessed using a mixed linear effects model implemented in MATLAB with percent-fibrosis as the dependent variable, patient as the random effect, and AP properties, clinical parameters, and electrophysiological tissue abnormalities (see above) as fixed effects.

The association of clinical parameters, electrophysiological tissue abnormalities, and fibrosis (as independent variables) with the occurrence of clinical arrhythmias (*i.e.* arrhythmias seen in patients *in vivo*) and with the occurrence of tissue arrhythmias (as dependent variables) was evaluated by binomial logistic regressions using OriginPro 2021 software (OriginLab Corporation, Northampton, MA, USA). Comparison of AP parameters (RMP, APA, dV/dt_max_, APD_20_, APD_50_, APD_90_, AUC_90_) in tissue with drug-induced arrhythmias and in tissue with spontaneous arrhythmias was performed by two-sample t-test in OriginPro 2021 software.

A *p*-value < 0.05 was considered as indicating a statistically significant difference for all analyses. In the manuscript, the estimate, *i.e.* the difference between each level mean and the overall mean, is reported for all mixed linear effects model results. All values are given as mean ± standard error of the mean, unless indicated otherwise.

# Supplemental Figures


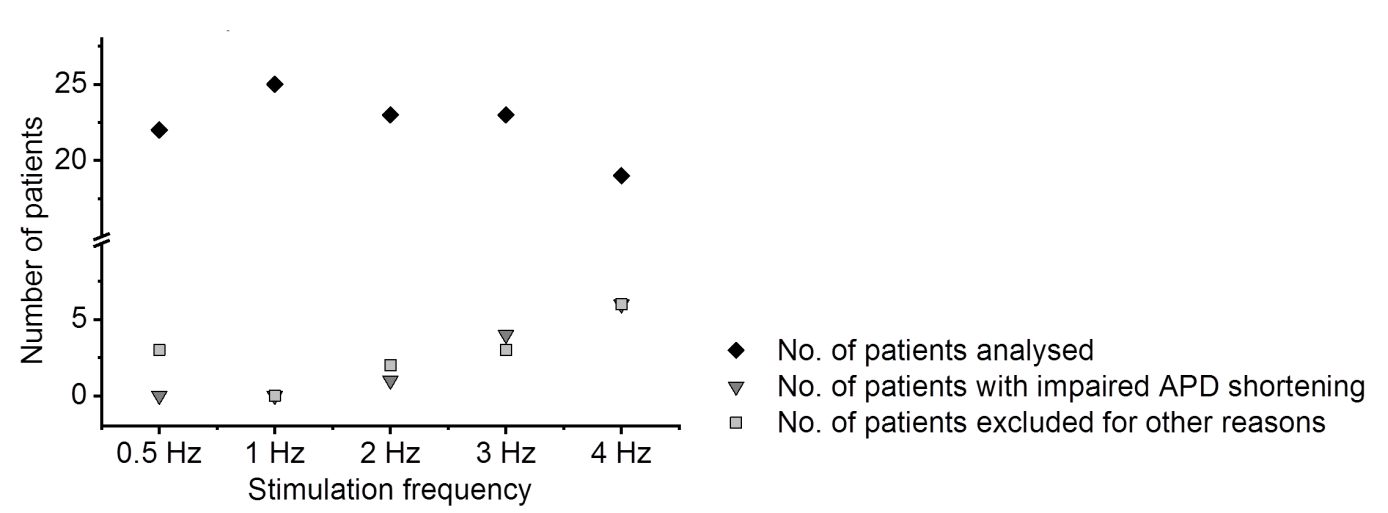


**Fig. S1 Number of patients included in the action potential analysis.** Exclusion was necessary either due to impaired action potential duration shortening with increasing stimulation frequency, artefacts in the recording, or technical issues. *APD* action potential duration.


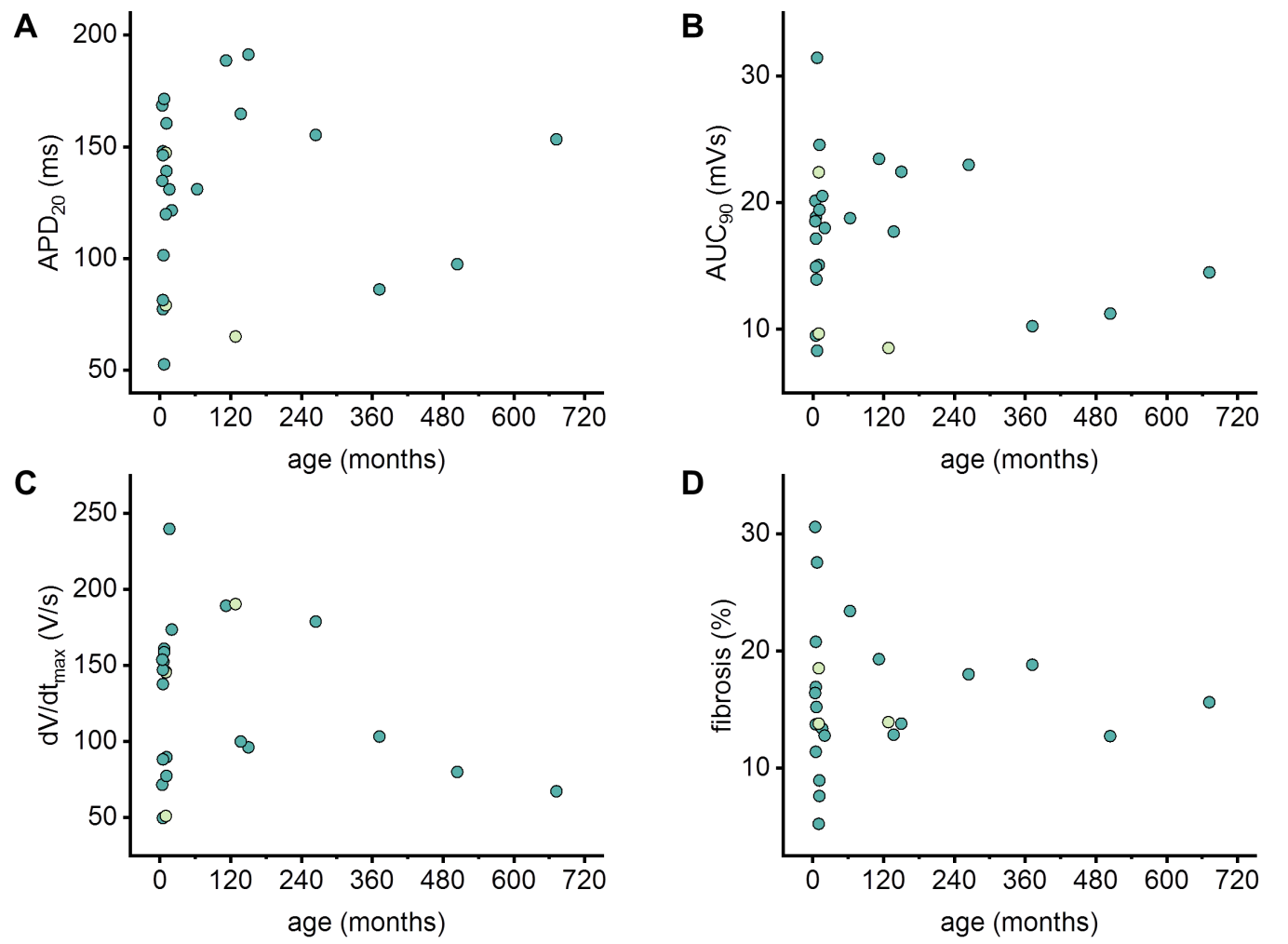


**Fig. S2 Effect of age on action potential shape at 1 Hz stimulation frequency and on fibrosis.** **A−D** Despite significant relation of age with action potential duration at 20% repolarisation, area under the curve at 90% repolarisation, maximum upstroke velocity, and percent-fibrosis (est. −1.6 ms, *p* = 0.022; est. 1.5 mV*s, *p* < 0.001; est. 0.2 V/s, *p* = 0.001; and est. −0.7%, *p* = 0.010, respectively) interpretation of biological relevance is limited due to the low number of data points at higher age. One data point represents one patient; patients with tetralogy of Fallot are presented in dark green, patients with atrial septal defect in light green. *APD_20_* action potential duration at 20% repolarisation, *AUC_90_* area under the curve at 90% repolarisation, *dV/dt_max_* maximum upstroke velocity.

# Supplemental Tables

**Table S1 Action potential parameters averaged across patients at different stimulation frequencies, including patients with impaired action potential duration shortening.** *N*= 25. Data shown are mean ± standard error of the mean.

|  | **Stimulation frequency** | | | | |
| --- | --- | --- | --- | --- | --- |
|  | 0.5 Hz | 1 Hz | 2 Hz | 3 Hz | 4 Hz |
| RMP (mV) | −72.1 ± 1.0*^ns^* | −71.8 ± 1.1 | −71.8 ± 0.7*^ns^* | −71.1 ± 1.0*^ns^* | −72.0 ± 1.6*^ns^* |
| APA (mV) | 92.5 ± 1.8*^ns^* | 91.8 ± 1.6 | 91.1 ± 1.3*^ns^* | 89.6 ± 1.7^**^ | 87.8 ± 1.8^***^ |
| dV/dt_max_ (V/s) | 145.6 ± 12.1*^ns^* | 127.1 ± 10.3 | 117.9 ± 8.3^*^ | 105.7 ± 8.3^**^ | 97.3 ± 6.7^***^ |
| APD_20_ (ms) | 144 ± 10.1^***^ | 129 ± 7.8 | 108 ± 6.8^***^ | 88 ± 5.4^***^ | 71 ± 5.8^***^ |
| APD_50_ (ms) | 229 ± 16.3^***^ | 202 ± 12.4 | 169 ± 10.6^***^ | 136 ± 9.0^***^ | 100 ± 7.0^***^ |
| APD_90_ (ms) | 299 ± 21.0^***^ | 269 ± 16.2 | 231 ± 15.2^***^ | 183 ± 8.1^***^ | 148 ± 8.4^***^ |
| AUC_90_ (mV*s) | 19.5 ± 1.5^***^ | 17.3 ± 1.2 | 14.5 ± 0.9^***^ | 11.1 ± 0.6^***^ | 8.5 ± 0.6^***^ |

Asterisks indicate significant difference of given parameter and frequency compared to 1 Hz (linear coefficient test on the mixed model coefficient ‘frequency’; ****p* < 0.001, ***p* < 0.01, **p*< 0.05, *ns* not significant). *APA* action potential amplitude, *APD* action potential duration, *AUC* area under the curve, *dV/dt_max_* maximum upstroke velocity, *RMP* resting membrane potential.

**Table S2** **Action potential parameters averaged across patients at different stimulation frequencies, excluding those patients with impaired action potential duration shortening.** *N* = 19. Data shown are mean ± standard error of the mean.

|  | **Stimulation frequency** | | | | |
| --- | --- | --- | --- | --- | --- |
|  | 0.5 Hz | 1 Hz | 2 Hz | 3 Hz | 4 Hz |
| RMP (mV) | −72.1 ± 1.3 | −71.8 ± 1.3 | −71.7 ± 0.9 | −71.8 ± 1.4 | −72.5 ± 2.0 |
| APA (mV) | 92.2 ± 2.2 | 90.9 ± 1.9 | 90.8 ± 1.4 | 89.7 ± 1.9 | 89.7 ± 2.0 |
| dV/dt_max_ (V/s) | 157.8 ± 12.3 | 125.5 ± 12.3 | 123.4 ± 8.7 | 114.3 ± 8.7 | 103.9 ± 6.6 |
| APD_20_ (ms) | 127 ± 9.8 | 116 ± 8.1 | 96 ± 7.3 | 80 ± 5.9 | 61 ± 4.5 |
| APD_50_ (ms) | 200 ± 14.3 | 179 ± 11.5 | 148 ± 9.1 | 121 ± 9.1 | 92 ± 6.2 |
| APD_90_ (ms) | 269 ± 17.1 | 237 ± 13.0 | 201 ± 10.9 | 171 ± 8.3 | 138 ± 7.1 |
| AUC_90_ (mV*s) | 17.0 ± 1.4 | 15.2 ± 1.0 | 12.6 ± 0.8 | 10.3 ± 0.6 | 8.0 ± 0.5 |

*APA* action potential amplitude, *APD* action potential duration, *AUC* area under the curve, *dV/dt_max_* maximum upstroke velocity, *RMP* resting membrane potential.

**Table S3 Detailed clinical patient information, electrophysiological tissue abnormalities, and percent-fibrosis for each of the study patients.**

|  | Pre-operative clinical parameters | | | | | | | | | | Tissue parameters | | | |
| --- | --- | --- | --- | --- | --- | --- | --- | --- | --- | --- | --- | --- | --- | --- |
| Pat. no. | Gender | CHD | Age (mo.) | Repair status | QRS dur.^a^ | Cyanosis | RV-PA grad. (mmHg) | β-bl. therapy | ProBNP (pg/ml) | Clinical arr.^b^ | Tissue arr. | Imp. APD short. | APD altern. | fibrosis (%) |
| 1 | male | TOF | 150 | unrep | normal | no | 86 | no | 49 | SVT | no | at 3 Hz | yes | 13.77 |
| 2 | male | TOF | 7 | unrep | normal | yes | 81 | no | 420 | atrial flutter | no | no | no | n/a |
| 3 | male | TOF | 112 | rep | 140 ms | no | 38 | no | 644 | no | EAD | at 3 Hz | yes | 19.30 |
| 4 | female | TOF | 4 | unrep | normal | yes | 38 | metopr | 497 | no | no | no | no | 16.40 |
| 5 | male | TOF | 5 | unrep | normal | yes | 80 | propran | 870 | no | no | no | yes | 13.72 |
| 6 | female | TOF | 5 | unrep | normal | no | 21 | metopr | 1088 | no | no | no | no | 11.36 |
| 7 | female | TOF | 16 | unrep | normal | yes | 52 | propran | 267 | ectopic atrial tachyc. | EAD | no | yes | 13.34 |
| 8 | male | TOF | 20 | unrep | normal | no | 91 | propran | 77 | no | EAD | no | yes | 12.76 |
| 9 | male | TOF | 10 | unrep | normal | yes | 17 | metopr | 508 | no | no | no | no | 5.21 |
| 10 | female | TOF | 11 | unrep | normal | yes | 99 | propran | 280 | no | no | at 2 Hz | no | 8.92 |
| 11 | female | TOF | 137 | unrep | normal | no | 106 | no | 49 | no | EAD | no | yes | 12.83 |
| 12 | male | ASD | 128 | unrep | normal | no | 6 | no | 91 | no | no | no | no | 13.90 |
| 13 | female | ASD | 10 | unrep | normal | no | 3 | no | 400 | no | EAD | no | no | 13.78 |
| 14 | male | TOF | 11 | unrep | normal | yes | 91 | no | 1406 | SVT | no | at 4 Hz | yes | 7.59 |
| 15 | female | TOF | 6 | unrep | normal | no | 61 | no | 158 | accel. junct. rhythm | SD/C | no | no | 15.20 |
| 16 | male | ASD | 10 | unrep | normal | no | 5 | no | 64 | no | no | no | no | 18.51 |
| 17 | male | TOF | 672 | rep | 160 ms | no | 11 | no | 51 | no | no | no | yes | 15.61 |
| 18 | female | TOF | 5 | unrep | normal | yes | 98 | no | 234 | no | SD/C | no | yes | 20.78 |
| 19 | male | TOF | 504 | rep | 160 ms | no | 14 | no | 157 | no | EAD | no | no | 12.71 |
| 20 | female | TOF | 63 | rep | 100 ms | yes | 9 | no | 660 | no | EAD, SD/C | no | yes | 23.41 |
| 21 | male | TOF | 7 | unrep | normal | no | 80 | metopr | 497 | no | EAD | at 3 Hz | no | 27.57 |
| 22 | male | TOF | 372 | rep | 200 ms | no | 9 | no | 107 | no | no | no | yes | 18.82 |
| 23 | male | TOF | 5 | unrep | normal | no | 42 | no | 459 | no | no | no | no | 16.91 |
| 24 | male | TOF | 4 | unrep | normal | no | 73 | propran | 458 | AV dissoc. | SD/C | no | no | 30.60 |
| 25 | male | TOF | 264 | rep | 180 ms | no | 7 | no | 120 | no | extrasyst. | at 4 Hz | yes | 18.00 |

^a^Absolute QRS duration only given for prolonged values (reference values for paediatric and for adult patients from ^2,3^ [references 27 and 28 in the manuscript]). ^b^All clinical tachyarrhythmias occurred in the immediate post-operative phase only; there was no manifestation of ventricular tachyarrhythmias or history of pre-operative tachyarrhythmias in the study cohort. *Accel. junc. rhythm* accelerated junctional rhythm, *ASD* atrial septal defect, *altern.* alternans, *APD* action potential duration, *arr.* arrhythmia, *AV* *dissoc*. atrio-ventricular dissociation, *β-bl. therapy* beta blocker therapy, *CHD* congenital heart defect, *dur.* duration, *EAD* early afterdepolarisation, *electrophys.* electrophysiological, *extrasyst.* extrasystoles, *imp. APD shorten.* impaired APD shortening with increasing stimulation frequency, *metopr* metoprolol, *mo.* months, *pat. no.* patient number, *proBNP* pro brain natriuretic peptide, *rep* repaired, *RV-PA grad.* echocardiographically measured pressure gradient between right ventricle and pulmonary artery, *SD/C* spontaneous depolarisations in the form of couplets, *SVT* supraventricular tachycardia, *TOF* tetralogy of Fallot, *unrep* unrepaired.

# References

1. Wülfers EM, Greiner J, Giese M, et al. Quantitative collagen assessment in right ventricular myectomies from patients with tetralogy of Fallot. *Europace*. 2021;23(Suppl 1):i38-i47. doi:10.1093/europace/euaa389
2. Davignon A, Rautaharju P, Boisselle E, Soumis F, Mégélas M, Choquette A. Normal ECG standards for infants and children. *Pediatr Cardiol*. 1980;1(2):123-131. doi:10.1007/BF02083144
3. Surawicz B, Childers R, Deal BJ, Gettes LS. AHA/ACCF/HRS Recommendations for the standardization and interpretation of the electrocardiogram. *Circulation*. 2009;119(10):e235-e240. doi:10.1161/CIRCULATIONAHA.108.191095
